# A High-Performance Lossless Compression Scheme for EEG Signals Using Wavelet Transform and Neural Network Predictors

**DOI:** 10.1155/2012/302581

**Published:** 2012-02-29

**Authors:** N. Sriraam

**Affiliations:** Center for Biomedical Informatics and Signal Processing and Department of Biomedical Engineering, SSN College of Engineering, SSN Nagar, Kalavakkam, Chennai 603 110, India

## Abstract

Developments of new classes of efficient compression algorithms, software systems, and hardware for data intensive applications in today's digital health care systems provide timely and meaningful solutions in response to exponentially growing patient information data complexity and associated analysis requirements. Of the different 1D medical signals, electroencephalography (EEG) data is of great importance to the neurologist for detecting brain-related disorders. The volume of digitized EEG data generated and preserved for future reference exceeds the capacity of recent developments in digital storage and communication media and hence there is a need for an efficient compression system. This paper presents a new and efficient high performance lossless EEG compression using wavelet transform and neural network predictors. The coefficients generated from the EEG signal by integer wavelet transform are used to train the neural network predictors. The error residues are further encoded using a combinational entropy encoder, Lempel-Ziv-arithmetic encoder. Also a new context-based error modeling is also investigated to improve the compression efficiency. A compression ratio of 2.99 (with compression efficiency of 67%) is achieved with the proposed scheme with less encoding time thereby providing diagnostic reliability for lossless transmission as well as recovery of EEG signals for telemedicine applications.

## 1. Introduction

Medical signal processing is a fast growing field of research that is producing increasingly sophisticated applications in today's high-tech medicine [1–8]. In the field of neurology, EEG, the manifestation of brain's electrical activity as scalp potentials, remains as one of the commonly used noninvasive techniques for understanding brain functions in health and disease. Since its discovery by Berger [9], many research activities have centered on how to automatically extract useful information about the brain's conditions based on the distinct characteristics of EEG signals. Many applications require acquisition, storage, and automatic processing of EEG during an extended period of time [4, 10–20]. For example, 24 h monitoring of a multiple-channel EEG is needed for epilepsy patients. The frequency range of a normal adult EEG lies between 0.1–100 Hz; thus, a minimum sampling rate of 200 Hz is needed. At the quantization level of 16 bit/sample, a 10-channel EEG for a 24 h period would require storage space of 346 Mb. Furthermore in order to diagnose the disease and to assess the effectiveness of the treatment via the brain functions, the analysis process normally takes a very long period of time. Since every sample of EEG signal is important and cannot be neglected without consultation of experts, legal storage of long-term EEG signals has to be done without any loss.

 The compression of Electroencephalographic (EEG) signal is of great interest to many in the biomedical community [4, 10–20]. The motivation for this is the large amount of data involved in collecting EEG information which requires more memory for storage and high bandwidth for transmission. Lossless compression of EEG is essential due to the necessity for exact recovery of the data for diagnostic purposes [10]. Efficient compression of the EEG signal is a difficult task due to the randomness inherent in the signal. This makes it difficult to obtain high compression rates with lossless compression methods [10]. An excellent review of compression techniques applied to EEG signals has been reported in [10]. Two-stage lossless compression schemes involving predictors and entropy encoders have been reported in [4, 10–14, 21–23]. In [4, 22], context- based offset bias cancellation has been applied to the predictive error to improve the distribution of residues suitable for encoding. Agarwal and Gotman [15] discussed EEG compression schemes for intensive care applications. Yi et al. [16] proposed an adaptive bit assignment algorithm to compress the raw EEG signal. Sriraam and Eswaran proposed an adaptive error modeling scheme which replaces the encoder in the second stage [17] and it has been shown that histogram-based region selection by heuristic search improves the compression efficiency.

**Figure 1:**
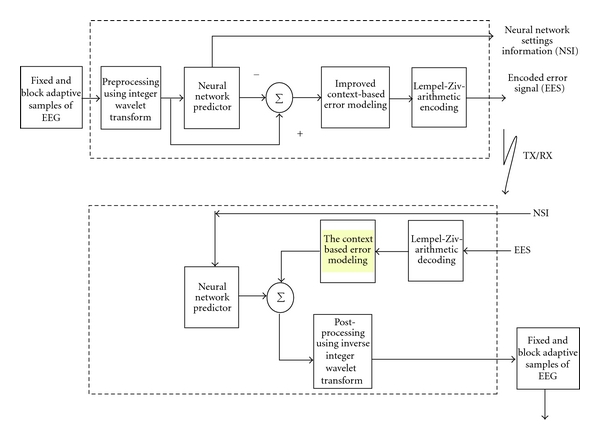
Proposed lossless compression scheme.

 In [18] Wongsawat et al. applied the Karhunen-Loeve transform (KLT) for lossless EEG compression. The effect of uniform quantization on near-lossless compression of EEG signals has been reported by the author [19]. Gopikrishna and Makur discussed a near-lossless compression scheme using wavelets and ARX model [20]. Lossy compression based on genetic algorithm, wavelet-packets, and neural network and linear predictors have been reported [21–23]. Recent works reported based on pursuit approach with wavelet dictionaries, wavelet-SPIHT, and finite rate of innovation technique exploiting sampling theory have shown some improvement in the compression performance [24–26]. It has been observed from the existing literature that even though several compression techniques have been reported, the search for new methods continues to achieve higher compression efficiency, while preserving the point-to-point diagnostic information in the reconstructed signal. This paper highlights a high performance lossless EEG compression using wavelet transform and neural network predictors. Even though the combinations of wavelet and neural network have been reported for compression problems [27–29], it has not been extensively applied for 1D biomedical signals.  Figure 1 shows the proposed lossless EEG compression scheme.

The coefficients generated by integer wavelet transform are used to train the neural network predictors. The error residues obtained as the difference between the actual and the predicted wavelet coefficient are further encoded using a combinational entropy encoder, Lempel-Ziv-arithmetic encoder (LZARIC). The “nsi” carries the initial setting information such as predictor order, weights, and so forth to set up the counterpart network at the receiving end. Lossless compression is assured due to the fact that the decoded error signal with the predicted signal recovers the original signal without losing any diagnostic information. Three neural network models, namely, single-layer perceptron (SLP), multilayer perceptron (MLP), and Elman network (EN) are used as predictors [13, 14, 21–23] and the performance are compared with adaptive linear predictors such as normalized least mean-square FIR and AR model [4, 30, 31]. EEG signals recorded under different physiological conditions are considered.

 Two training schemes, namely, single block (SB) and block adaptive (BA) training schemes are used for training the neural network predictors [30, 32] and the performance of the proposed lossless scheme is evaluated in terms of bits per sample (BPS) and compression ratio (CR). In order to improve the compression efficiency without losing any inherent information, an improved context-based error modeling is also investigated. This paper is organized as follows.  Section 2 presents a brief introduction to preprocessing using wavelets transform and the neural network-based prediction.  Section 3 discusses the improved context-based error modeling. The proposed high performance computing algorithm is tested using EEG signals recorded under different physiological conditions, its compression results, computational complexity and compression with other coding scheme in the literature are presented in Section 4. Finally Section 5 provides the concluding remarks of the paper.

## 2. Preprocessing and Predcition

### 2.1. Wavelet-Based Preprocessing

**Figure 2:**
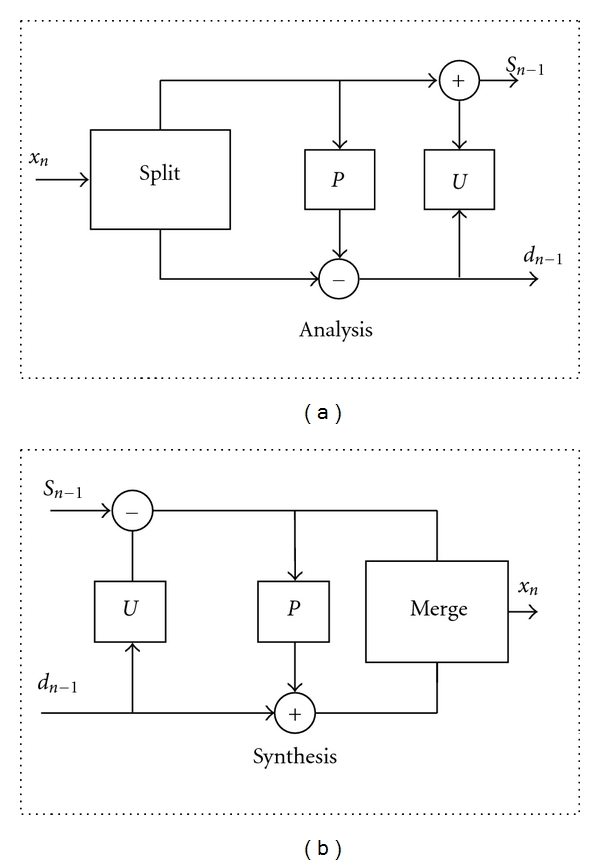
Forward and inverse lifting scheme.

Compression techniques based on the wavelet decomposition of the 1D and 2D digital signals have received much attention due to its excellent energy compaction capabilities [3, 17, 19, 33–38] as well as its ability to locate the short-time high frequency features of a signal and at the same time ability to resolve low-frequency behavior [38]. The wavelet lifting transform proposed by Sweldens [39] which allows fast, efficient, and in place calculation of the wavelet coefficients provides the feasibility to reconstruct the integer wavelets thereby satisfying the lossless criterion [37, 39].  Figure 2 shows the forward and inverse wavelet lifting transform [27, 37, 39]. The detail operation of the lifting approach of wavelet transform are reported in [37, 39].

### 2.2. Neural Network Predictors

Lossless compression using neural network predictors is achieved, when it simulates identical prediction processes [13, 14, 21–23, 32, 40]. The characteristics of neural networks such as massive parallel structure, high degree of interconnection, capabilities of high-speed computation, nonlinear mapping, and self-organization makes it best candidate for prediction and compression problems [40]. In this work, three neural network models, single-layer and multilayer perceptrons, a feedforward model, and Elman network feedback model are considered [13, 14, 21–23]. The SLP network consists of only an input layer and an output layer with no hidden layer. All input nodes or neurons including the bias nodes are connected to all output nodes. The MLP consists of a number of interconnected layers of independent neurons where the outputs of one layer form the inputs to next layer. MLP consists of three layers, namely, the input layer, the hidden layer, and the output layer. Elman networks (ENs) are two-layer backpropagation networks, with the addition of a feedback connection from the outputs of the hidden layer to its input [23, 41]. The feedback allows Elman networks to learn to recognize and generate temporal patterns as well as spatial patterns [23, 41]. The Elman network differs from conventional two-layer network in that the first layer has a recurrent connection [23, 41]. The delay in this connection stores values from the previous time step, which can be used in the current time step. In order to compare the performance of neural network predictors, two linear predictors, namely, autoregressive model (AR) [4] and normalized least-mean square adaptive finite impulse response filter (FIR) [30, 31] are considered. The predicted sample,$\;{\hat{x}}_{n}$ related to the current wavelet coefficient sample *x*_*n*_ is shown in

(1) $${\hat{x}}_{n}=f\left(\overset{p}{\underset{i=1}{\sum }}\left(x{}_{n\text{–}1}w_{i}\right)\right),$$

where *w* stands for the set of all the connection weights, “*p*” is the order of the predictor.

Using the criterion of minimizing the mean squared error *E*[*e* ^2^(*n*)] as given in (2), one can calculate the error between the actual and target samples:

(2) $$\begin{array}{cc} \underset{w}{\min}\;E\left[e^{2}\left(n\right)\right] & =\underset{w}{\min}\;E\left[{\left(x\left(n\right)-\hat{x}\left(n\right)\right)}^{2}\right] \end{array}.$$

## 3. Improved Context-Based Error Modeling

Context-based error modeling has gained much research importance in improving the performance of compression algorithms [4, 22, 42, 43]. It is known that most state-of-the- art lossless coding techniques comprise prediction, context modeling of prediction error followed by entropy encoding [42]. The prediction error sequence is a composition of multiple sources of varying statistics of distributed different means. Context modeling of prediction error is a means to separate these distributions thereby adjusting the offset to yield zero Laplacian distribution. Such scheme is referred to as context-based bias cancellation [4, 22, 42, 43] and has been applied to EEG signals using linear predictors [4] and neural network predictors [22]. In [4, 22] contexts were framed by computing the difference between the adjacent two samples. An improved context calculation is introduced in this work, where the context for each sample is computed by determining the difference between the past sample and next sample as shown in (3). In this way, entropy coding efficiency increases marginally

(3) $$d_{i}=x_{k+i}-x_{k-i},\;1\leq i\leq m,$$

where “*m*” is any integer value.

The signal pattern is well exploited through the formation of contexts that help in better distribution of prediction errors [22, 23, 42, 43]. The application of appropriate encoder further decrease the bits required for transmitting the signal. In order to reduce the number of contexts, quantization of the *d*_*i*_ values can be performed [42, 43]. A simple quantization is to obtain a two-level value based on certain threshold as shown in

(4) $$Q\left(d_{i}\right)=\{\begin{array}{cc} 0, & d_{i}<0\\ 1, & d_{i}\geq 0 \end{array}$$

## 4. Expermental Evaluation and Results

### 4.1. EEG Datasets

**Figure 3:**
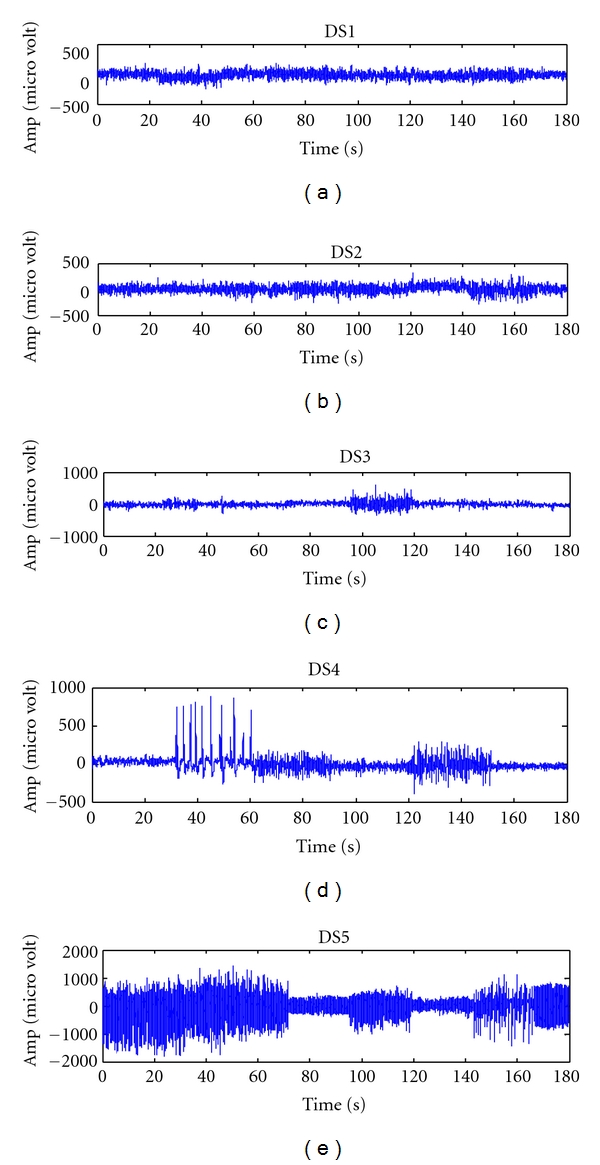
Sample recordings of DS1–DS5.

For experimental study, recordings of EEG grabbed from extracranial and intracranial electrodes obtained from the host site of Epileptology Department, University of Bonn are used [44]. Data sets 1 and 2 (DS1 and DS2) are obtained from healthy volunteers in, an awaken state with eyes open (DS1) and eyes closed (DS2), respectively which are recorded using surface electrodes [23, 33]. Data set (DS3) is extracted from hippocampal formation of the opposite hemisphere of the brain and Data set 4 (DS4) is recorded from within the epileptic zone [44]. DS3 and DS4 contained activity measured during seizure-free intervals. Data set 5 (DS5) contains recordings exhibiting ictal seizure activity. DS3–DS5 is recorded using intracranial electrodes [44]. A total of 15-minute recordings of EEG are considered. DS1–DS5 is represented with 12 bit accuracy with a sampling rate of 173.61 Hz [44].  Figure 3 shows the sample recordings of EEGs with 180 s samples.

### 4.2. Performance Evaluation

The compression performance of neural network predictor is evaluated in terms of the compression ratio (CR) which is defined as follows:

(5) $$\left(\text{CR}\right)=\frac{vn}{pn+wj+rk},$$

where *v*: total number of samples in test file, *n*: total number of bits used to represent a original sample, *p*: order of the predictor, *w*: number of bits to represent the wavelet coefficient, *j*: number of bits to represent a weight, *r*: total number of error residue samples, *k*: number of bits to represent the entropy encoded sample.

Two schemes, namely, single-block (SB) and block-adaptive (BA) schemes are used [45, 46] to process the input EEG signal. In SB scheme, the entire EEG signals are considered as a single fixed block. In BA scheme, EEG signals are divided into block size of 90 s samples. For preprocessing, 5/3 biorthogonal wavelet transform with four decomposition levels is used [27]. The wavelet coefficients are then used to train the neural network predictors. Before training the feedforward network, the weights are initialized using Nyuyen-Widrow algorithm [13, 41]. The order of the predictors (number of input neurons) chosen are 2, 5, 10, and 20, respectively. For the output layer, different activation function is used and the optimal function is identified. For MLP, several backpropogation learning rules are investigated and the optimal rule is identified [41]. The appropriate activation function for the hidden layer and output layer are also determined. The numbers of hidden neurons used for MLP are 1, 3, 8, and 12 with predictor orders 2, 5, 10, and 20, respectively. When EN is created, each layer's weight is initialized with the Nguyen-Widrow layer initialization [41]. For EN, several backpropogation learning rules are investigated and the optimal rule is identified [41]. The appropriate activation function for the hidden layer and output layer is also determined. The number of hidden neurons used for EN are 1, 5, 8, and 16 with predictor orders 2, 5, 10, and 20, respectively, are used. Levenberg-Marquart backpropagation learning algorithm is used for SLP and MLP and gradient descent with momentum and adaptive learning rate backpropagation algorithm is used for EN [17, 19].

**Figure 4:**
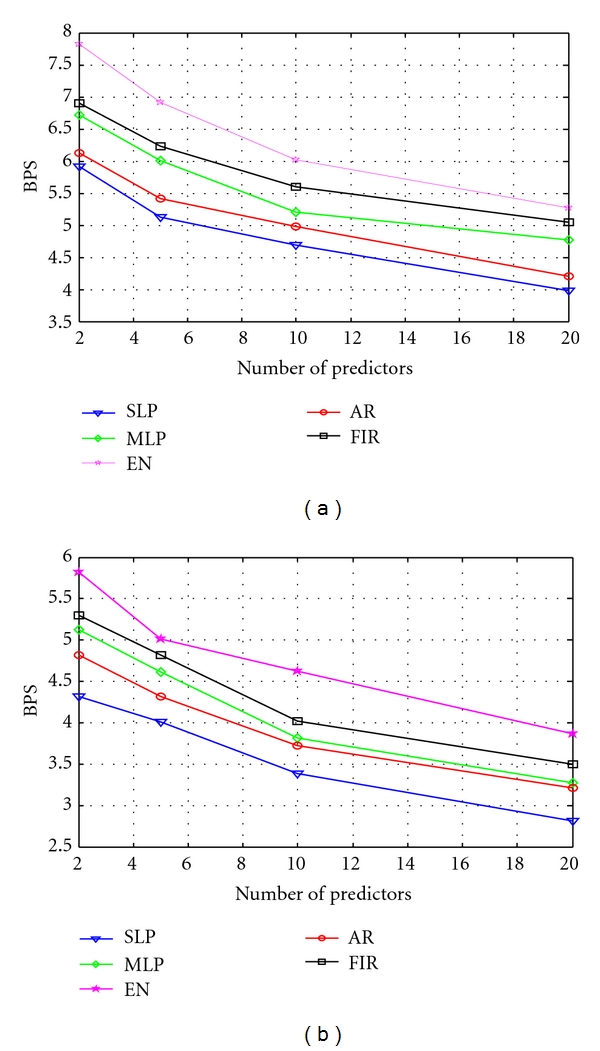
(a) Values of BPS obtained for SB scheme and (b) Values of BPS obtained for BA scheme.

For linear prediction, a fifth-order autoregressive (AR) model [4, 19, 23] and a fifth-order FIR [12, 19, 30, 31] are used. In order to estimate the AR parameters, Levinson-Durbin's method as reported in [4] is used. For improved context-based bias cancellation, number of contexts used are 4, 8, 16, 32, and 256, respectively, and compression performance is evaluated. Then the improved error residues obtained after error modeling are further encoded using combinational encoder, LZARIC. The reason for choosing this encoder is due to its ability to provide high compression efficiency compared to other entropy encoders [23, 47]. Figures 4(a) and 4(b) show the BPS value obtained using different predictors by varying the prediction order for the SB and BA schemes, respectively, with 32 contexts. The average values obtained using all the datasets, DS1–DS5, are only given.

**Figure 5:**
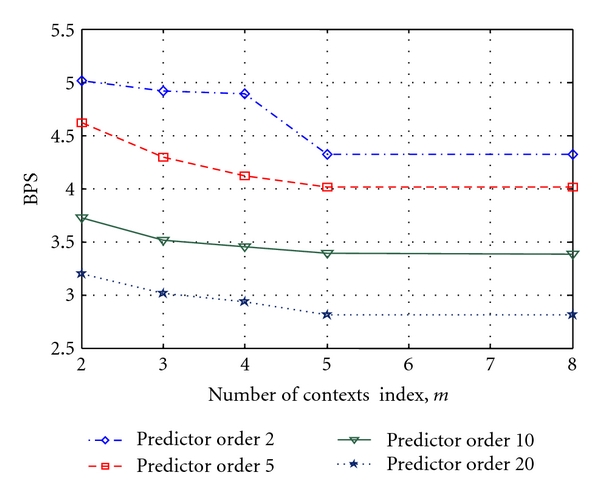
Values of BPS obtained using different contexts with prediction order for SLP-BC-LZARIC scheme.

It can be seen from Figures 4(a) and 4(b) that BA scheme requires less number of bits compared to SB scheme. Among the different predictors used, SLP yields the best compression results. The compression performances are also evaluated with different contexts and prediction order combinations for SLP and Figure 5 depicts the results.

It can be observed from Figure 5 that increase in context index until 5 decreases the value of BPS. Further increase in *m* = 5 does not yield significant improvement in BPS value. The performances of the proposed lossless scheme are evaluated in terms of CR with the following schemes:

- without applying bias cancellation (WBC),
- with bias cancellation (BC) [22],
- with improved bias cancellation (IBC).

**Figure 6:**
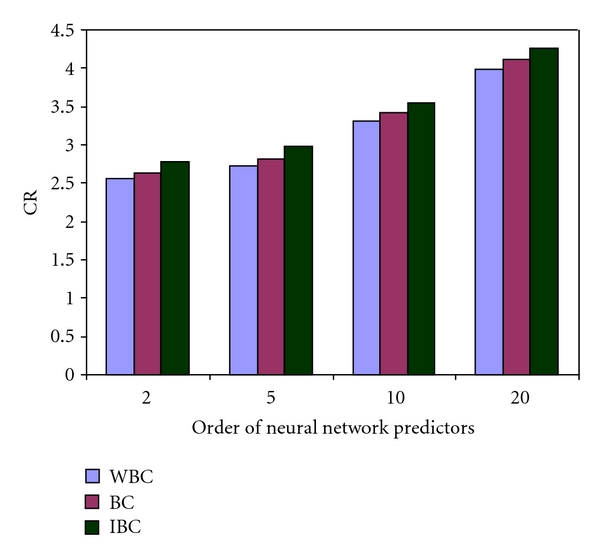
Compression performance with and without error modeling.

Figure 6 shows the value of CR obtained using SLP predictor with the above schemes followed by LZARIC encoder.

From Figure 6, it can be seen that the application of improved bias cancellation improves the compression performance with an average CR value of 0.14.

### 4.3. Computational Complexity

**Figure 7:**
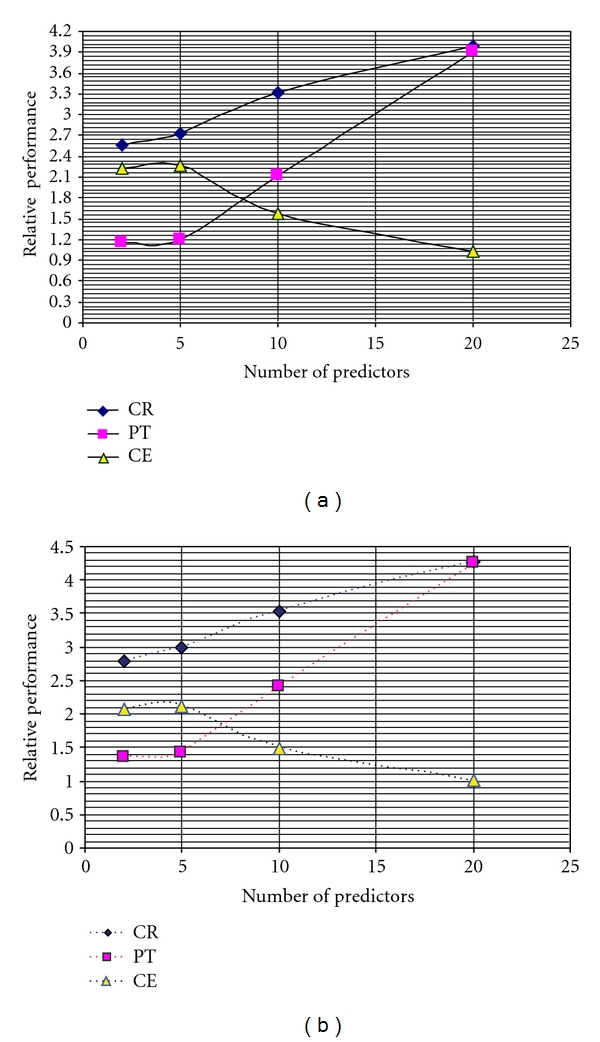
(a) Relative performances of the proposed compression scheme without error modeling. (b) Relative performances of the proposed compression scheme with error modeling.

The complexity of the compression system is the computational effort need for encoding and decoding the EEG signal. The total processing time required per sample either at the transmission end (encoder) or at the receiving end (decoder) is based on the sum of the contribution relative to the individual block shown in Figure 1. Figures 7(a) and 7(b) show the relative performance of our proposed compression scheme in terms of processing time calculated (PT) in seconds, CR, and computational efficiency (CE) [17, 23]. The computational efficiency (CE) as defined in [17] is given by

(6) $$\text{CE}=\frac{\text{CR}}{\text{Processing}\;\text{time}}.$$

It can be observed from Figures 7(a) and 7(b) that the fifth-order prediction yields the optimal results in terms of achieving best computational efficiency. Further the incorporation of error modeling does not degrade the CE value. The compression performance of the proposed scheme is compared with other known schemes reported in the literature [4, 10–12, 14, 18, 22]. Since there is no standard benchmark EEG data were available, an exact comparison cannot be performed.

Table 1 shows the values of CR obtained using the lossless schemes reported earlier as well as the proposed lossless scheme discussed in this work.

It can be noticed that our proposed scheme yields good compression results with compression efficiency [19] of 67% compared to the results reported earlier. Further from Figure 7, it can be seen that our scheme requires less encoding time which indicates its potential suitability for real-time transmission. Further the proposed scheme found to be better than the schemes reported recently [24–26].

## 5. Concluding Remarks

A high performance lossless compression has been discussed for EEG signals. The scheme involves preprocessing using integer wavelet transform, prediction using neural network predictors followed by adjusting the offset of the prediction residue through improved context-based bias cancellation. A Lempel-Ziv-arithmetic combinational encoder has been used to further encode the residues obtained from wavelet coefficients. EEG signals recorded under different physiological conditions have been used and data are segmented into single block and block adaptive for further training. Three neural network models, namely, single-layer perceptron, multilayer perceptron and Elman network have been used as predictors and the performance were compared with adaptive linear predictors such as normalized least mean-square FIR and AR model. The performances of the proposed scheme were evaluated in terms of bits per sample (BPS). It has been found from the experimental results that the application of wavelets and improved context error modeling improves the compression efficiency. The adaptive error modeling scheme as reported in [17] can very well replace the combinational encoder discussed in this work to further improve the compression efficiency.

## Figures and Tables

**Table 1:** Comparison of compression results.

Vector quantization with collapsed Huffman tree [1]	RLSL filters/ High pass filter with arithmetic coding [2]	Dynamical neural network with arithmetic coding [3]	Linear predictor (AR) with error modeling and Huffman coding [8]	SLP predictor with error modeling and arithmetic coding [22]	KLT transform [18]	Wavelet-SPIHT [26]	Proposed scheme
2.63	1.61	2.08	2.50	2.72	2.84	2.046	2.99
